# Inferring ethnicity from mitochondrial DNA sequence

**DOI:** 10.1186/1753-6561-5-S2-S11

**Published:** 2011-05-28

**Authors:** Chih Lee, Ion I Măndoiu, Craig E Nelson

**Affiliations:** 1 Computer Science and Engineering Department, University of Connecticut, Storrs, CT, USA; 2 Molecular and Cell Biology Department, University of Connecticut, Storrs, CT, USA

## Abstract

**Background:**

The assignment of DNA samples to coarse population groups can be a useful but difficult task. One such example is the inference of coarse ethnic groupings for forensic applications. Ethnicity plays an important role in forensic investigation and can be inferred with the help of genetic markers. Being maternally inherited, of high copy number, and robust persistence in degraded samples, mitochondrial DNA may be useful for inferring coarse ethnicity. In this study, we compare the performance of methods for inferring ethnicity from the sequence of the hypervariable region of the mitochondrial genome.

**Results:**

We present the results of comprehensive experiments conducted on datasets extracted from the mtDNA population database, showing that ethnicity inference based on support vector machines (SVM) achieves an overall accuracy of 80-90%, consistently outperforming nearest neighbor and discriminant analysis methods previously proposed in the literature. We also evaluate methods of handling missing data and characterize the most informative segments of the hypervariable region of the mitochondrial genome.

**Conclusions:**

Support vector machines can be used to infer coarse ethnicity from a small region of mitochondrial DNA sequence with surprisingly high accuracy. In the presence of missing data, utilizing only the regions common to the training sequences and a test sequence proves to be the best strategy. Given these results, SVM algorithms are likely to also be useful in other DNA sequence classification applications.

## Introduction

Human ethnic identity is a controversial and complex topic. Each human individual is a complex mosaic of genetic material originating from a multitude of ancestral sources. However, despite this complexity, the division of humans into coarse ethnic groupings can greatly assist forensic investigators and is also increasingly being used as a predictor of drug effectiveness in the emerging fields of personalized medicine and race-based therapeutics. Self-reported and investigator-assigned ethnicity typically rely on the subjective interpretation of a complex combination of both genetic and non-genetic information including behavior, cultural and societal norms, skin color, and other influences. For this reason, attempts to accurately infer probable coarse ethnic identity can be difficult in contexts with limited access to most informative markers, such as skin and hair samples. In these situations genetic information can be extremely valuable to forensic pursuits by significantly enhancing the accuracy of coarse ethnic classification in these contexts.

Several approaches to genetic-based inference of ethnicity have been proposed in the literature. In particular, the use of panels of autosomal markers have been shown to provide excellent accuracy for assigning samples to specific clades [1,2]. Unfortunately, these approaches rely on typing large numbers of autosomal loci that may not survive long periods of degradation. Mitochondrial DNA, however, due to its high-copy number, is recoverable even from minute or highly degraded samples. Furthermore, due to its high polymorphism and maternal inheritance, mitochondrial DNA has proved to be an excellent marker for the inference of ethnic affiliation. Indeed, several studies including [3-5] have previously shown the feasibility of inferring the probable ethnicity and/or geographic origin from the sequence of the hypervariable region (HVR) of the mitochondrial genome. These studies clearly demonstrate that, although the mitochondrial sequence alone does not by itself determine one’s ethnicity, the two are nevertheless strongly associated.

In this paper we test the utility and robustness of several methods for the classification of HVR mitochondrial sequences into coarse ethnic groups as previously assigned by investigators from the FBI, self-assigned by study subjects, or by anthropologists. The goal was to identify a method that could most accurately reproduce these classifications using only a small region of the mitochondrial genome. As Egeland et al. [5], we consider a supervised learning approach to ethnicity inference. In this setting, mtDNA sequences with annotated ethnicity are used to “train” a classification function that is then used to assign ethnicities to new mtDNA sequences. Adopting this approach allows us to draw on the large body of knowledge developed within the machine learning community (see, e.g., [6]). The main goal of the paper is to assess the performance of four well-known classification algorithms (support vector machines, linear discriminant analysis, quadratic discriminant analysis, and nearest neighbor) on a variety of benchmark datasets including realistic levels of missing data and training data bias.

Comprehensive experiments conducted on mtDNA profiles extracted from the mtDNA population database [7] show that the support vector machine algorithm is the most accurate of compared methods, outperforming both discriminant analysis methods previously employed in [3-5]) as well as a nearest neighbor algorithm similar to that used for haplogroup inference in [8]. In both cross-validation and experiments conducted on independently collected training and test data, SVM achieves an overall accuracy of 80-90%, matching the accuracy of human experts making ethnicity assignments based on physical measurements of the skull and large bones [9,10], and coming close to the accuracy achieved by using approximately sixty autosomal loci [11]. These results demonstrate that SVM effectively classifies sequences from a small segment of the mitochondrial genome and that these classifications can be used to predict the probable assignment of coarse ethnicity with reasonable accuracy. The superiority of SVM in this classification problem suggests that it is also likely to be superior in similar sequence classification applications.

## Methods

In this section, we introduce the four methods of ethnicity assignment investigated in this study and the datasets used to evaluate their empirical performance. We begin by briefly introducing principal component analysis (PCA), a dimensionality reduction technique used as a preprocessing step for three of the four methods. We then describe the four classification algorithms – support vector machines (SVM), linear discriminant analysis (LDA), quadratic discriminant analysis (QDA) and 1-nearest neighbor (1NN). Finally, we describe the datasets used for evaluation, the conversion of mtDNA sequence profiles into feature vectors, and methods of encoding sequences with missing regions.

### Principal component analysis

PCA (see [6] for an introduction) is a factor analysis technique of dimensionality reduction. Given *m* samples over *n* variables, the *m* samples can be represented as a *m* × *n* matrix **X**. We further assume that the sample mean of each variable is 0, that is,  for every *j*. Projecting the *m* samples onto *n* new axes yields another *m* × *n* matrix **Y** = **XP**, where **P** is a *n* × *n* orthogonal matrix whose columns are unit vectors defining the *n* new axes. PCA finds a **P** such that the sample covariance matrix of the *n* new variables is a diagonal matrix, that is,(1)

where **D** is a diagonal matrix, and **Σ_X_** and **Σ_Y_** are the sample covariance matrices of the original and new variables, respectively. The orthogonal matrix **P** can be easily obtained by eigenvalue decomposition of **Σ_X_**. PCA is a dimensionality reduction technique in that only *k* of the *n* new variables are kept for further analysis. A standard approach is to pick the *k* variables with the largest sample variances. Therefore, all we need to do is to pick the value of *k.* Fortunately, when PCA is used in conjunction with supervised learning algorithms like classification algorithms, the best value of *k* can be selected by performing cross-validation. In this study, *k* was selected by performing 5-fold cross-validation (CV) on the training data for each combination of dataset and classification algorithm.

### Classification algorithms

#### Support vector machines

The SVM [12] is a binary classification algorithm. In the case of perfectly separable classes, SVM seeks a separating hyperplane with maximum margin, while for non-separable classes the goal is to maximize a linear combination of the separation margin and the total amount by which SVM predictions fall on the wrong side of their margin. Given *n*-element feature vectors **x*_i_***, *i* = 1,…, *m*, and an *m*-element label vector **y** such that *y_i_* ? {1, –1}, this amounts to solving the following optimization problem:(2)

where *C* > 0 is a penalty constant, *ξ_i_* is the slack variable allowing misclassification of sample *i*, *Φ*(⋅) is a function that maps ***x****_i_* to a high-dimensional space, often called the feature space, and ***β***, *β*_0_ define the optimum separating hyperplane ***β***^T^***z*** + *β*_0_ = 0 in feature space. Once the optimal separating hyperplane is found, a test sample ***t*** is classified according to the sign of ***β***^T^*Φ*(***t***) + *β*_0_.

In practice, the solution to the convex optimization problem (2) is obtained by solving the so-called Wolfe dual. Instead of explicitly mapping samples to the feature space, solving the dual requires only a kernel function K(***x***_1_,***x***_2_) = *Φ*(***x***_ι_)^T^*Φ*(***x***_2_), which implicitly maps samples to the feature space and simultaneously computes the inner product [12]. In this study, we used the software package LIBSVM [13] to conduct all SVM experiments. LIBSVM uses the “one-against-one” approach [14] when more than two classes are present. For all SVM experiments we used the radial basis kernel K(***x***_1_,***x***_2_) = exp(*-γ|**x***_1_*-**x***_2_|^2^), where *γ* is a parameter. The penalty constant *C* and the parameter *γ* were tuned using 5-fold cross-validation on the training data.

#### Linear and quadratic discriminant analysis

LDA and QDA assume that for each class the feature vectors follow a multivariate normal distribution [6]. That is, the conditional probability of a sample ***x*** given that it belongs to class *g* is given by(3)

By applying Bayes’ theorem, we obtain the posterior distribution as follows.(4)

where *π_g_* is the prior probability of class *g.* The parameters of the multivariate normal distribution are estimated using the training dataset. LDA assumes that the classes have a common covariance matrix (i.e., **Σ***_g_* = **Σ** for every *g*) therefore fewer parameters need to be estimated for LDA compared to QDA. For both methods, a given test sample ***t*** is assigned to the class with the highest posterior probability

argmax*_g_* Pr(*G* = *g|****X*** = ***t***)*.*

In this study, we used MCLUST Version 3 [15] to conduct all LDA and QDA experiments.

#### 1-nearest neighbor (1NN)

1NN is a simple non-parametric classification algorithm, which does not have a training process. Given a set of reference samples and a test sample, 1NN searches the reference dataset for the sample nearest to the test sample and assigns the test sample to the class to which the nearest sample belongs. In case there are multiple nearest reference samples, voting is used to assign the test sample to the class containing the largest number of nearest reference samples. As discussed below, mtDNA profiles are encoded into binary feature vectors. We used the number of mismatch positions (a.k.a. the Hamming distance) to measure the distance between samples, and did not apply PCA to the data before applying 1-NN.

### Datasets

We used the forensic and published tables in the mtDNA population database [7] to empirically evaluate the performance of the four algorithms for ethnicity assignment. The forensic table contains 4,839 samples collected and typed by the Federal Bureau of Investigation (FBI), while the published table contains 6,106 samples collected from the literature.

In this study, we focus only on the samples annotated as belonging to one of the four coarse ethnic groups – Caucasian, African, Asian and Hispanic. Filtering the forensic and published tables by this criteria results in 4,426 and 3,976 samples, respectively. In the rest of the paper we will refer to the two filtered tables simply as the *forensic* and *published datasets*. The forensic dataset contains 1,674 Caucasian (37.8%), 1,305 African (29.5%), 761 Asian (17.2%) and 686 Hispanic (15.5%) samples, while the published dataset is comprised of 2,807 Caucasian (70.6%), 254 African (6.4%) and 915 Asian (23%) samples.

Additional file 1 shows the percentage of samples sequenced at each position for the forensic and published datasets. We note that the forensic dataset has a significantly better coverage than the published dataset. All the samples in the forensic dataset cover portions of both hypervariable region 1 (HVR1) and hypervariable region 2 (HVR2) of mtDNA, whereas over 60% of samples in the published dataset do not cover HVR2 and around 5% of them do not cover HVR1.

To better characterize and compare the forensic and published datasets, we assign each sample in the two datasets to one of the 23 basal haplogroups defined in [8]. Haplogroup assignment was performed using the unweighted 1NN algorithm described in [8] along with the Genographic Project open resource mitochondrial DNA database (the consented database) of 21,164 samples [16]. Behar et al. [8] reported a leave-one-out cross-validation accuracy of 96.72% on a reference database of 16,609 samples. We observed a comparable accuracy of 96.51% on the consented database. Therefore, we expect the inferred haplogroups of samples in the forensic and published datasets to have a similarly high accuracy. The ethnicity composition of each haplogroup and the inferred haplogroup composition of each broad ethnic group represented in the forensic and published datasets are given in Additional file 2. Additional file 2(A) supports the well known fact that many haplogroups are strongly associated with a specific ancestry. For example, most samples with inferred haplogroup H, J, K, R0*, T, U*, and V are Caucasian, most samples with inferred haplogroup B, D, M, N, and R9 are Asian, and most samples with inferred haplogroup L are African. However, the association is not perfect, and significant percentages of these haplogroups are present in other ethnic groups. For some haplogroups, such as B, N1*, W, and X the association with ethnicity is particularly weak, with two or three ethnicities being represented in almost equal proportions. Additional file 2 further shows that the forensic and published datasets have significant differences in their ethnic and haplogroup compositions. Most strikingly, Caucasians are significantly over-represented and Hispanics are completely missing from the published dataset. Such differences are most likely due to the procedure used to assemble the published dataset, and reflects preferential use of samples from some ethnic groups in published studies.

For some of the experiments described in the Results section, we used specific subsets of the forensic and published datasets. The *full-length forensicdataset* consists of the 1,904 samples typed for the most extensive ranges of HVR1 (16024–16569) and HVR2 (1–576). This dataset is comprised of 222 Caucasian (11.7%), 820 African (43.1%), 415 Asian (21.8%) and 447 Hispanic (23.5%) samples. The *trimmed forensic dataset* was produced by trimming the samples in the forensic dataset such that only the region of 16024–16365 in HVR1 is kept. It has the same ethnicity composition as the forensic dataset since all samples in the forensic dataset are typed in this range. The *trimmed publisheddataset* was created in a similar fashion, except that only 2,540 samples covering the 16024-16365 region were kept. This subset contains 1,956 Caucasian (77%), 134 African (5.3%) and 450 Asian (17.7%) samples.

### Encoding mtDNA profiles into feature vectors

Each sample in the forensic and published datasets is given as a list of polymorphic changes when compared to the revised Cambridge Reference Sequence (rCRS). For example, 16298C denotes a substitution at position 16298 and 16124.1C denotes the insertion of a C after position 16124. For a fixed dataset, we represent each sample as an *n*-element binary vector, where *n* is the number of unique polymorphisms present in the dataset. An element in the binary vector of a sample is set to 1 if the sample harbors the corresponding polymorphism, and to 0 otherwise. This encoding method works well when all the samples in the dataset are sequenced over the same or very similar ranges. An example is the forensic dataset, in which all samples cover range 16024-16365 of HVR1 and range 73-340 of HVR2. While most of our experiments were obtained using the above binary encoding, we also discuss and evaluate in the Results section several alternative schemes for encoding mtDNA profiles with significant amounts of missing data.

## Results

### Comparison of the four classification algorithms

For an initial evaluation of the four classification algorithms, we performed cross-validation (CV) analysis using the trimmed forensic dataset. Cross-validation is one of the simplest and most widely used methods for estimating the accuracy of classification algorithms. Briefly, available samples are randomly split into K roughly equal parts, and then each part is used to evaluate classification accuracy of a model trained on the remaining *K* – 1 parts. In our experiments we used *K* = 5, i.e., 5-fold cross-validation.

In addition to ethnicity-wise average accuracies, we also use *micro*- and *macro-accuracy* as measures of the overall performance of the classification algorithms. These metrics, similar to the micro-average and macro-average of [17], are defined as follows:(5)(6)

where *K* is the number of classes in the dataset, *N_i_* is the number of samples in class *i* and *C_i_* is the number of samples correctly labeled by the classifier in class *i*. Note that micro- and macro-accuracy become the same when classes sizes are balanced, i.e., *N*_1_ = *N*_2_ = ⋯ = *N_K_*. For imbalanced class sizes, micro-accuracy tends to over-emphasize the performance on the largest classes compared to macro-accuracy, which gives equal weight to the accuracy achieved for each class.

Table 1 summarizes the 5-fold CV accuracy metrics for PCA-QDA, PCA-LDA, 1NN, and PCA-SVM on the trimmed forensic dataset. PCA-SVM consistently outperforms the other three classification algorithms with respect to all accuracy measures. Since the performance of different classification algorithms may depend significantly on the typed mtDNA region, we conducted three additional experiments to assess its effect on the classification accuracy of the four compared algorithms. In all three of them we started from the full-length forensics dataset. In the first experiment, we iteratively deleted 10% of the polymorphisms, starting from the HVR2 end non-adjacent to HVR1. Similarly, in the second experiment, we iteratively deleted 10% of the polymorphisms starting from the HVR1 end non-adjacent to HVR2. Finally, in the third experiment, we used a sliding window approach to generate 20 different datasets, each of which retained from the full-length forensics profiles 10% of the nucleotides.

**Table 1:** Comparison of 5-fold CV accuracy measures on the trimmed forensic dataset

	# Samples	Classification Algorithm			
		
		PCA-QDA	PCA-LDA	1NN	PCA-SVM
**Caucasian**	1674	83.15	90.2	93.73	94.62

**Asian**	761	72.93	74.11	83.31	84.76

**African**	1305	84.6	88.28	86.59	89.81

**Hispanic**	686	71.57	68.22	72.01	72.59

**Micro-Accuracy**	4426	80.03	83.46	86.47	88.10

**Macro-Accuracy**	4426	78.06	80.20	83.91	85.45

**Figure 1:**
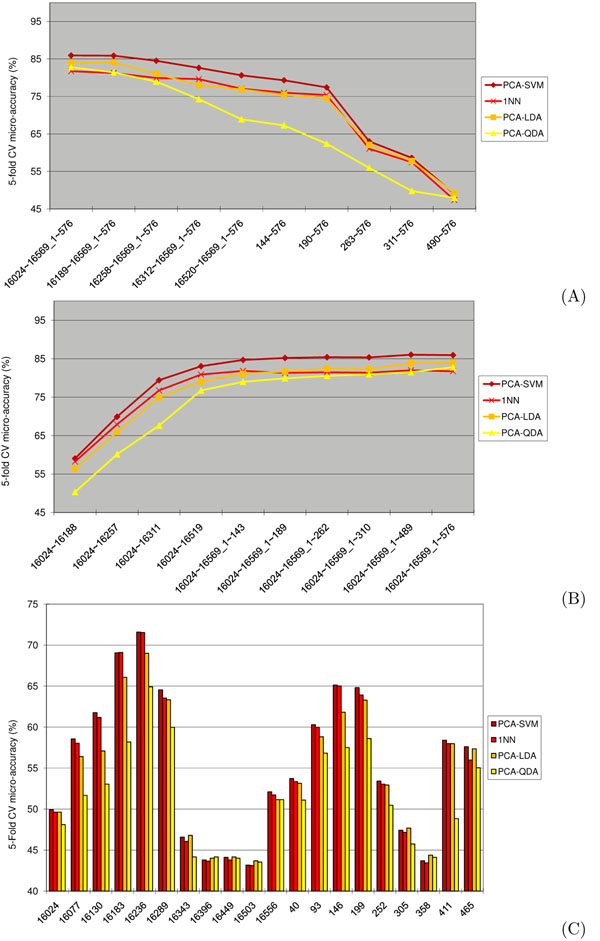
**Effects of incomplete data on accuracy** Comparison of PCA-QDA, PCA-LDA, 1NN, and PCA-SVM 5-fold CV micro-accuracy on regions obtained by iteratively deleting groups of 10% polymorphisms starting from HVR1 towards HVR2 (A), respectively from HVR2 towards HVR1 (B), and on sliding windows spanning 10% of the nucleotides in HVR1+HVR2 (C).

Figure 1 gives the 5-fold CV micro-accuracy achieved by PCA-QDA, PCA-LDA, 1NN, and PCA-SVM in these three experiments. Again, PCA-SVM consistently outperforms the other three classification algorithms investigated in this study. PCA-QDA is typically outperformed by the other methods, except that it outperforms 1NN when the entire HVR is used. 1NN and PCA-LDA have comparable performance, but PCA-LDA performs slightly better than 1NN for near-complete mtDNA profiles. Conversely, 1NN performs better than PCA-LDA for some short typed regions. Indeed, for short windows consisting of only 10% of the nucleotides in the entire dataset, the performance of 1NN is often as good as that of PCA-SVM, see Figure 1(C).

Figure 1(C) further shows that, regardless of the classification method used, certain regions of HVR1 and HVR2 are more informative than others for the purpose of ethnicity inference. Additional file 3 gives the 5-fold CV micro-accuracy for 6 selected windows of 165-271bp spanning the most informative regions of HVR1 and HVR2. Interestingly, when using about 200bp from the information-rich region of HVR1, PCA-SVM yields a microaccuracy of over 80%, very close to the microaccuracy achieved on this set when using the entire HVR region, i.e., HVR1+HVR2.

### Validating SVM on independent test data

Cross-validation may overestimate the practical performance of classifiers since it ignores potentially significant biases in the assembly of reference databases. To obtain a more reliable estimate for the practical accuracy of PCA-SVM, we evaluated its performance using the trimmed forensic dataset as training data and the trimmed published dataset as test data. Table 2 gives the so called confusion table for this experiment. There is no “Hispanic” row since there are no samples annotated as Hispanic in the trimmed published dataset used for testing. Since the Hispanic samples are present in the trimmed forensic dataset used for training, test samples may be mis-classified as Hispanic, and thus we do include a “Hispanic” column. PCA-SVM micro-accuracy, as well as ethnicity-wise accuracies for the Caucasian and African ethnic groups are similar to the cross-validation results in Table 1. However, ethnicity-wise accuracy for the Asian group is almost 17% lower than the accuracy achieved in the cross-validation experiment. This is largely explained by large mismatches between Asian profiles used for training and testing in this experiment. The 761 Asian profiles in the Forensic dataset used for training come from only 5 countries: China (356 profiles), Japan (163), Korea (182), Pakistan (8), and Thailand (52), with a strong bias towards East Asia. Not surprisingly, a large percentage of misclassifications errors (90 out of the total of 145) are for profiles collected from two countries (Kazakhstan and Kyrgyzstan) that are not represented in the training dataset. Profiles with unknown country of origin are also poorly classified (10 errors out of 22 samples) suggesting that they may come from regions that are poorly represented in the forensics dataset too.

**Table 2:** Confusion table of the PCA-SVM test results on the trimmed published dataset

True Ethnicity	# Samples	Predicted Ethnicity			
		
		Caucasian	Asian	African	Hispanic
**Caucasian**	1956	**92.59**	5.47	1.53	0.41

**Asian**	450	25.78	**67.78**	3.11	3.33

**African**	134	5.22	3.73	**87.31**	3.73

Micro-Accuracy: 87.91%					

Macro-Accuracy: 82.56%					

### Comparison of methods for handling missing data

In practice, forensic mtDNA profiles are determined by Sanger sequencing of PCR amplicons that span hypervariable regions HVR1 and HVR2. Different laboratories use different PCR primer pairs, some of which amplify only parts of HVR1 and HVR2. Quality trimming of Sanger chromatograms further results in confident polymorphism calls for a (sample dependent) subinterval of each amplicon. The end result are mtDNA profiles with a variable degree of sequence coverage, i.e., with unknown polymorphism status for some parts of HVR1 and/or HVR2. In the experiments reported in previous sections we relied on training and test sequences covering essentially the same range, so missing data was not an issue. In this section we reassess the accuracy of PCA-SVM under more realistic levels of missing data. Specifically, we report results of experiments performed using as training and test data the (untrimmed) forensic and published datasets, respectively; as shown in Additional file 1, the published dataset has indeed highly non-uniform coverage of different HVR regions.

We investigated three different approaches of dealing with missing data:

• **rCRS.** In this approach we simply assume that missing regions are identical to the rCRS. While easy to implement, this scheme is likely to introduce a strong bias towards the Caucasian ethnicity since the rCRS sequence is of a Caucasian.

• **Probability.** In this approach we augment the feature encoding scheme described in the Methods section by adding a set of *l* additional variables, where *l* is the total length of HVR1 and HVR2 in bases. For typed bases, these variables hold the mutation status of the base – 1 if there is a polymorphism at this base and 0 otherwise. For bases that are not covered by sequencing, the corresponding variable is set to a fractional value between 0 and 1 representing the polymorphism rate observed at this position in the training data. While less biased than the rCRS scheme, this scheme may still introduce unwanted biases in case some ethnicities are over- or under-represented in the training data.

• **Common region.** In this approach we compute, for each test profile, the intersection between the region sequenced in the test profile and each training sample. Only these common regions of the training sequences are then used to infer the ethnicity of the test sample. The common region approach is computationally more demanding than the other two, since it may require running PCA and training a new SVM for each test sample.

Additional file 4 summarizes the results obtained by using the three approaches to handling missing data in experiments in which the forensic and published datasets are used for training and evaluation classification accuracy, respectively. Consistent to its bias towards Caucasians, the rCRS approach has almost 97% accuracy for this ethnicity but very much lower accuracy for Asian and African ethnicities (about 31% and 59%, respectively), resulting in relatively poor overall micro- and macro-accuracies. The probability approach is still biased towards the Caucasian ethnicity, although less strongly than the rCRS approach. The best overall performance is achieved by the common region approach, which has micro- and macro-accuracies (as well as ethnicity-wise accuracies) very close to those observed in the experiments performed on the trimmed forensic and published datasets (see Table 2). This suggests that the common region approach is a good method of dealing with missing data, at least in conjunction with the PCA-SVM method for ethnicity inference.

A potential concern with using the common interval approach is that different amounts of training data are used in classifying different test samples. This can make it difficult to compare posterior probabilities returned by classification methods such as SVM, and may partly explain why, as shown in Additional file 5, SVM posterior probabilities typically under-estimate the observed accuracy.

## Discussion

### Correspondence between investigator assigned ethnicity and mitochondrial haplogroup

Human mitochondrial haplogroups have arisen from mutation and migration during human evolution. As such, these haplogroups have been extremely powerful tools in understanding human evolution and particularly in understanding patterns of geographical migration of human populations. Prior to modern travel, mitochondrial haplogroups were largely restricted to the geographic regions of their origin and subsequent migration. For this reason, they are often superimposed on maps of the globe as representative of the human populations derived from those regions of the planet. Similarly, but more crudely, the coarsest ethnic groupings of humans are also reflective of geographic ancestry. Africans, Caucasians, and Asians all have clear geographic associations, while Hispanic is often regarded as a less well defined mix of New World and European ancestry. Because of the clear associations of both mitochondrial haplogroups and ethnic categories with geography, one might naively expect a simple correlation between the two classifications. When we analyze the association between mitochondrial haplogroup and investigator assigned ethnicity however, we find a complex relationship between the two categories. While, for instance, there is broad correspondence between the L haplogroups and African ethnicity assignments, African ethnicity assignments are present to varying degrees in virtually every haplogroup analyzed and almost every haplogroup contains members of each of the four ethnicities. This is not particularly surprising due to the fact that mitochondrial DNA represents only a very small segment of the complex mosaic of a human’s genetic ancestry, and it suggests that the ability to infer coarse ethnic identity from mitochondrial sequence would be very limited. In fact, however, we find that mitochondrial DNA can be used to infer the probable assignment of coarse ethnicity with almost 90% accuracy, levels approaching those obtainable with approximately sixty autosomal loci [11]. This level of accuracy in predicting investigator assigned ethnicity could be very useful in forensic investigations.

### Information content in HVR1 and HVR2

As noted above, there is a great deal of variability in the precise regions of HVR1 and HVR2 genotyped in practice. Sequence coverage within the mitochondrial control region is often laboratory and/or study dependent. Variability of these boundaries severely limits the utility of individual datasets in the assembly of large datasets representative of complex populations. Recently, Tzen et al. [18] sought to redefine HVR1 on the basis of genetic diversity and laboratory tractability. They show that the 237-bp segment from 16126-16362 (the “redefined” HVR1, or rHVR1) had a global genetic diversity of 0.9905 and the 154-bp segment from 16209-16362 had a global diversity of 0.9735, where the genetic diversity for a sample with *n* haplotypes with population frequencies *x_i_*, *i* = 1,…,*n*, is computed as  . The results of [18] match very closely with our scans of the inferential power of windows across the control region; Tzen’s rHVR1 overlaps precisely with the region of greatest discriminative power in HVR1. The correspondence between these results suggests that HVR2 might be similarly standardized to a region between 93-310, where the greatest discriminative power of HVR2 is found. The identification of small regions of sequence that have maximal discriminative power could be quite useful in forensic and anthropological settings where severe degradation can limit the size of PCR products recoverable from sample material. Di Bernardo et al. [19] report that the longest amplifiable DNA fragments extracted from 2000-year-old remains from Pompeii are between 139 and 360 bp. Sequences of this size from the most informative regions of HVR1 and HVR2 would allow inference of coarse ethnic identity with reasonably high accuracy.

### SVM as classifier

Many applications in human genetics require the discriminative classification of samples into groups, and a number of methods for this task have been proposed. Lately, machine learning approaches have been used to good effect in a number of biological scenarios including the classification of Y-haplogroups [20]. In this study we use support vector machines (SVM) to develop statistical models capable of predicting the ethnicity of mitochondrial DNA samples. We compare the performance of SVM under simulations of real-world scenarios with several other methods previously proposed for the classification of mitochondrial sequences into geographically defined groups, including QDA and LDA [3-5]. In all tests SVM provides accuracy greater or equal to that of the other methods tested. SVM consistently provides the best accuracy in simulations of degradation form either end of the mitochondrial hypervariable regions, and when small subsections of the hypervariable regions are used. With only 218bp of mtDNA sequence, the overall accuracy of SVM predictions exceeds 80%. The success of SVM in this classification problem suggests that it may also be the best method for related classification problems including inferring the geographic origin of DNA samples [4,5], haplogroup membership [8], drug response profiles [21], and other “race based” therapeutics [22].

When applied to independent test data our SVM classifier performs reasonably well despite significant differences between the training and test sets. In particular, the absence of a Hispanic classification in the published dataset, and the inclusion of geographic regions in the test set that are not represented in the training set (for instance Kazakhstan and Kyrgyzstan) is likely to have contributed significantly to errors in our inferences. Such errors are likely to recede as larger, more geographically balanced training sets are assembled.

### Handling missing data

In the last few years several authors have pointed out the presence of sequence errors in public and forensic mtDNA databases [23-27]. Moreover, precise boundaries of HVR1 and HVR2 are not always consistent across studies and real-world samples may be severely degraded, further contributing to errors or missing data in samples to be classified. We evaluated several statistical approaches to dealing with missing data and evaluated these approaches for accuracy under simulated scenarios of data dropout or loss. We found that despite a small loss of accuracy incurred by data dropout, restricting analysis to the region of intersection between the test sample and training samples provides the most reliable inference of the ethnicity of the sample. Attempts to impute any missing data based on the rCRS or a probabilistic model based of the training set resulted in prediction bias toward Caucasian due to the origin of the rCRS and the preponderance of Caucasian samples in the FBI forensic data set. Until very large, ethnically balanced training sets are available, restricting analysis to the region of intersection between test and training samples is likely to remain the most accurate and unbiased approach to inference.

## Conclusions

In this study, we compared four classification algorithms for the prediction of probable assignment of coarse ethnic identity using short DNA sequences from the hypervariable region of mtDNA. Comprehensive empirical studies showed that, regardless of sequence length, support vector classification is the most accurate classifier among those compared and approaches 90% accuracy in predicting the assignment of course ethnic identity. Our experiments also identified high accuracy segments in HVR, which agree well with the genetically diverse regions reported in previous work. Finally, our experiments showed that, in dealing with missing data, it is advisable to use only segments shared by reference sequences and the sequence under test.

## Competing Interests

The authors declare that they have no competing interests.

## Authors contributions

IIM and CEN conceived the study. CL conducted the experiments. All the authors contributed valuable ideas to this study, drafted the manuscript, and were involved in manuscript revision. All authors have read and approved the final manuscript.

## Supplementary Material

Additional file 1 **Coverage of samples** Percentage of samples covering each position of HVR1 and HVR2 in the forensic (A) and published (B) datasets. Click here for file

Additional file 2 **Sample composition of the forensic and published datasets** Ethnicity composition of each haplogroup (A) and haplogroup composition of each ethnic group (B) for the forensic and published datasets. Click here for file

Additional file 3 **Accuracy of short segments of HVR** Comparison of PCA-QDA, PCA-LDA, 1NN, and PCA-SVM 5-fold CV micro-accuracy on 6 selected windows of 165-271bp spanning the most informative regions of HVR1 and HVR2. Click here for file

Additional file 4 Accuracy of PCA-SVM using different schemes for handling missing data Click here for file

Additional file 5 **Calibration of PCA-SVM posterior probabilities for the FBI published dataset** The actual accuracy rates are slightly higher than the estimated posterior probabilities. Click here for file
