# Supplementary material for: Inferring ethnicity from mitochondrial DNA sequence
Source: BMC Proc. 2011 May 28;5(Suppl 2):S11. doi: 10.1186/1753-6561-5-S2-S11 (PMC3090759; doi:10.1186/1753-6561-5-S2-S11)
Supplement: Additional file 1 — Coverage of samples Percentage of samples covering each position of HVR1 and HVR2 in the forensic (A) and published (B) datasets. [file 1753-6561-5-S2-S11-S1.pdf]

Additional file 1 — Coverage of samples

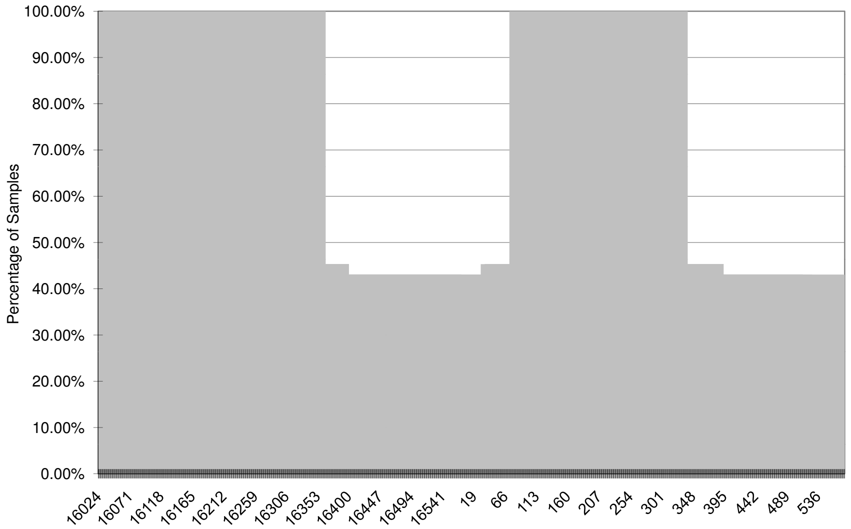

(A)

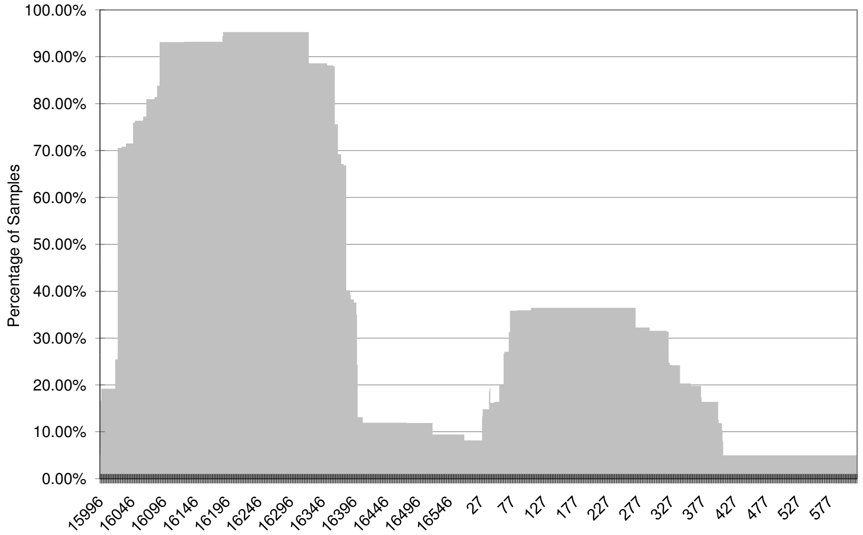

(B)

Percentage of samples covering each position of HVR1 and HVR2 in the forensic (A) and published (B) datasets.
