# Supplementary material for: Inferring ethnicity from mitochondrial DNA sequence
Source: BMC Proc. 2011 May 28;5(Suppl 2):S11. doi: 10.1186/1753-6561-5-S2-S11 (PMC3090759; doi:10.1186/1753-6561-5-S2-S11)
Supplement: Additional file 2 — Sample composition of the forensic and published datasets Ethnicity composition of each haplogroup (A) and haplogroup composition of each ethnic group (B) for the forensic and published datasets. [file 1753-6561-5-S2-S11-S2.pdf]

Additional file 2 — Sample composition of the forensic and published datasets

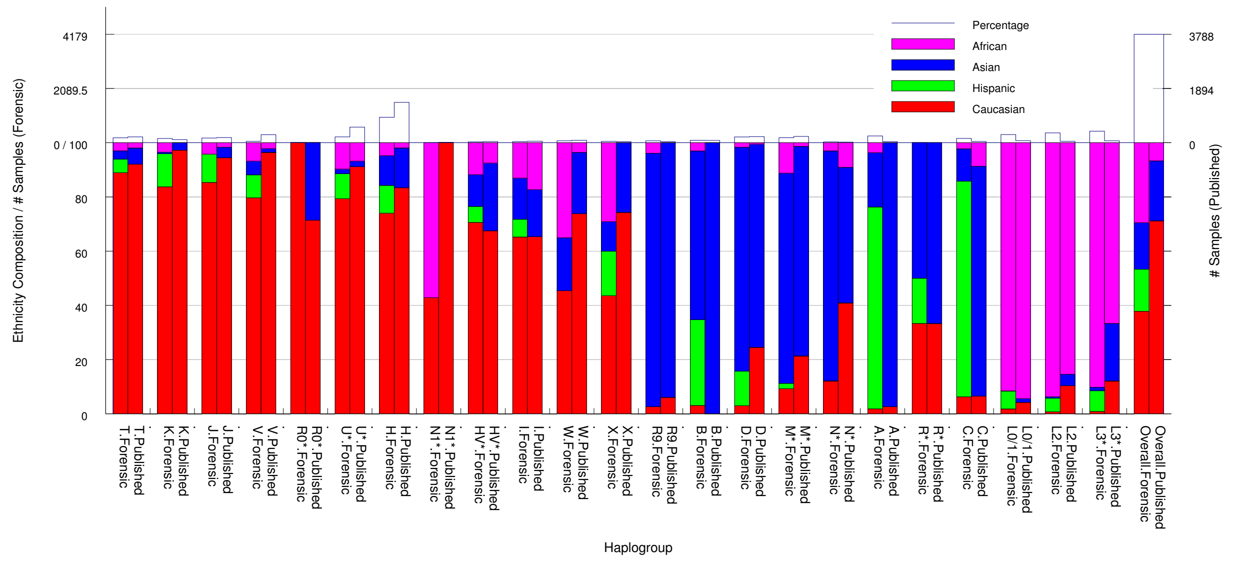

(A)

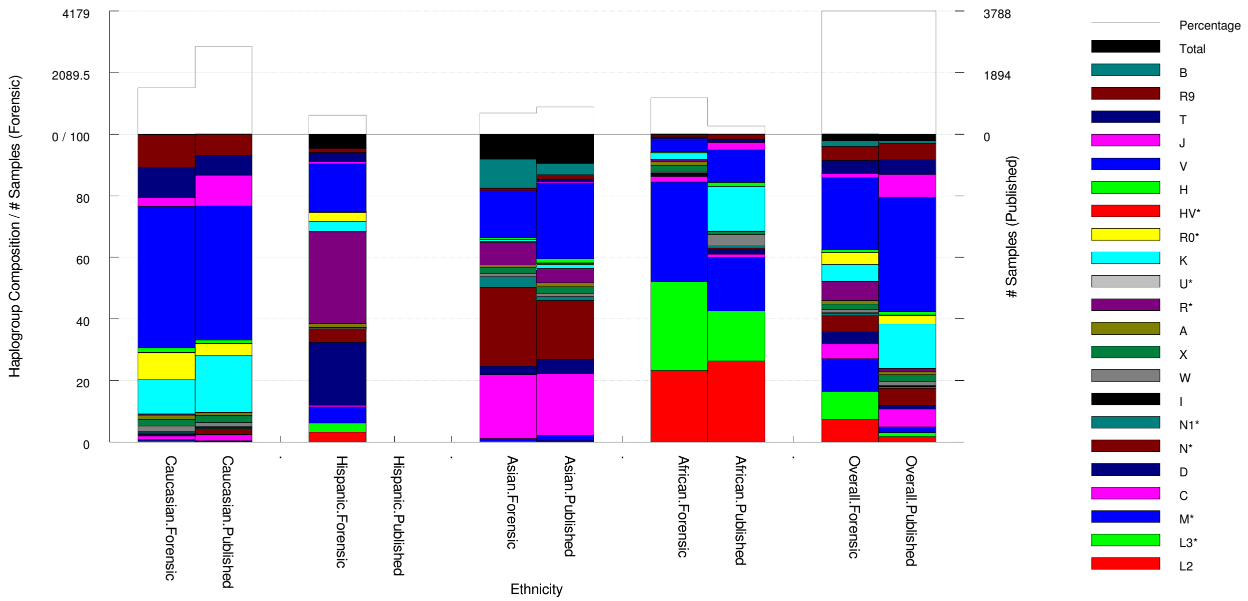

(B)

Ethnicity composition of each haplogroup (A) and haplogroup composition of each ethnic group (B) for the forensic and published datasets.
