# Supplementary material for: Inferring ethnicity from mitochondrial DNA sequence
Source: BMC Proc. 2011 May 28;5(Suppl 2):S11. doi: 10.1186/1753-6561-5-S2-S11 (PMC3090759; doi:10.1186/1753-6561-5-S2-S11)
Supplement: Additional file 3 — Accuracy of short segments of HVR Comparison of PCA-QDA, PCA-LDA, 1NN, and PCA-SVM 5-fold CV micro-accuracy on 6 selected windows of 165-271bp spanning the most informative regions of HVR1 and HVR2. [file 1753-6561-5-S2-S11-S3.pdf]

Additional file 3 — Accuracy of short segments of HVR

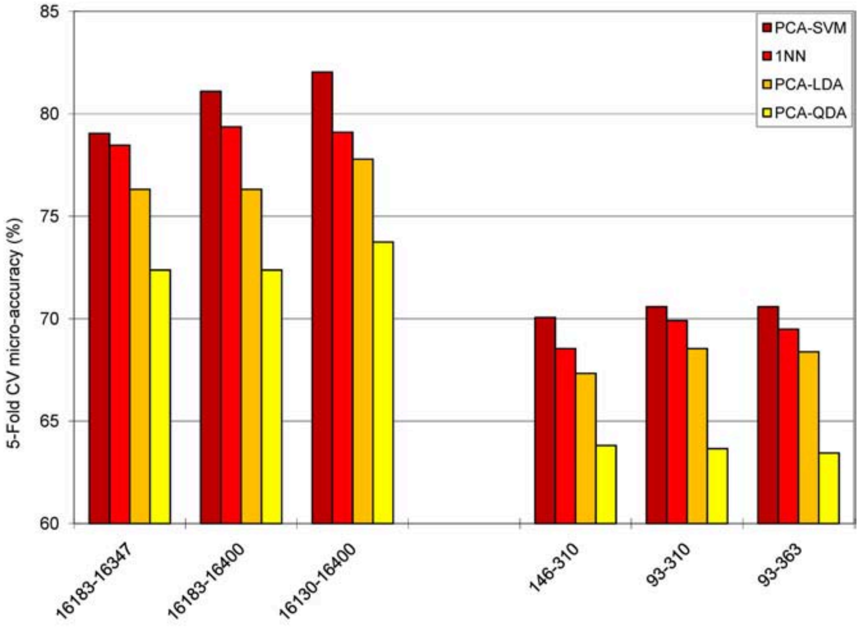

Comparison of PCA-QDA, PCA-LDA, 1NN, and PCA-SVM 5-fold CV micro-accuracy on 6 selected windows of 165-271bp spanning the most informative regions of HVR1 and HVR2.
