# Supplementary material for: Inferring ethnicity from mitochondrial DNA sequence
Source: BMC Proc. 2011 May 28;5(Suppl 2):S11. doi: 10.1186/1753-6561-5-S2-S11 (PMC3090759; doi:10.1186/1753-6561-5-S2-S11)
Supplement: Additional file 4 — Accuracy of PCA-SVM using different schemes for handling missing data [file 1753-6561-5-S2-S11-S4.pdf]

Additional file 4 — Accuracy of PCA-SVM using different schemes for handling missing data

Ethnicity	# Samples	Missing Data Handling		
		**rCRS**	**Probability**	**Common Region**
**Caucasian**	2807	96.94	96.15	93.94
**Asian**	915	30.60	50.93	68.85
**African**	254	59.06	57.48	76.38
**Micro-Accuracy**	3976	79.25	83.27	87.05
**Macro-Accuracy**	3976	62.20	68.19	79.72
