# Supplementary material for: Inferring ethnicity from mitochondrial DNA sequence
Source: BMC Proc. 2011 May 28;5(Suppl 2):S11. doi: 10.1186/1753-6561-5-S2-S11 (PMC3090759; doi:10.1186/1753-6561-5-S2-S11)
Supplement: Additional file 5 — Calibration of PCA-SVM posterior probabilities for the FBI published dataset The actual accuracy rates are slightly higher than the estimated posterior probabilities. [file 1753-6561-5-S2-S11-S5.pdf]

Additional file 5 — Calibration of PCA-SVM posterior probabilities for the FBI published dataset

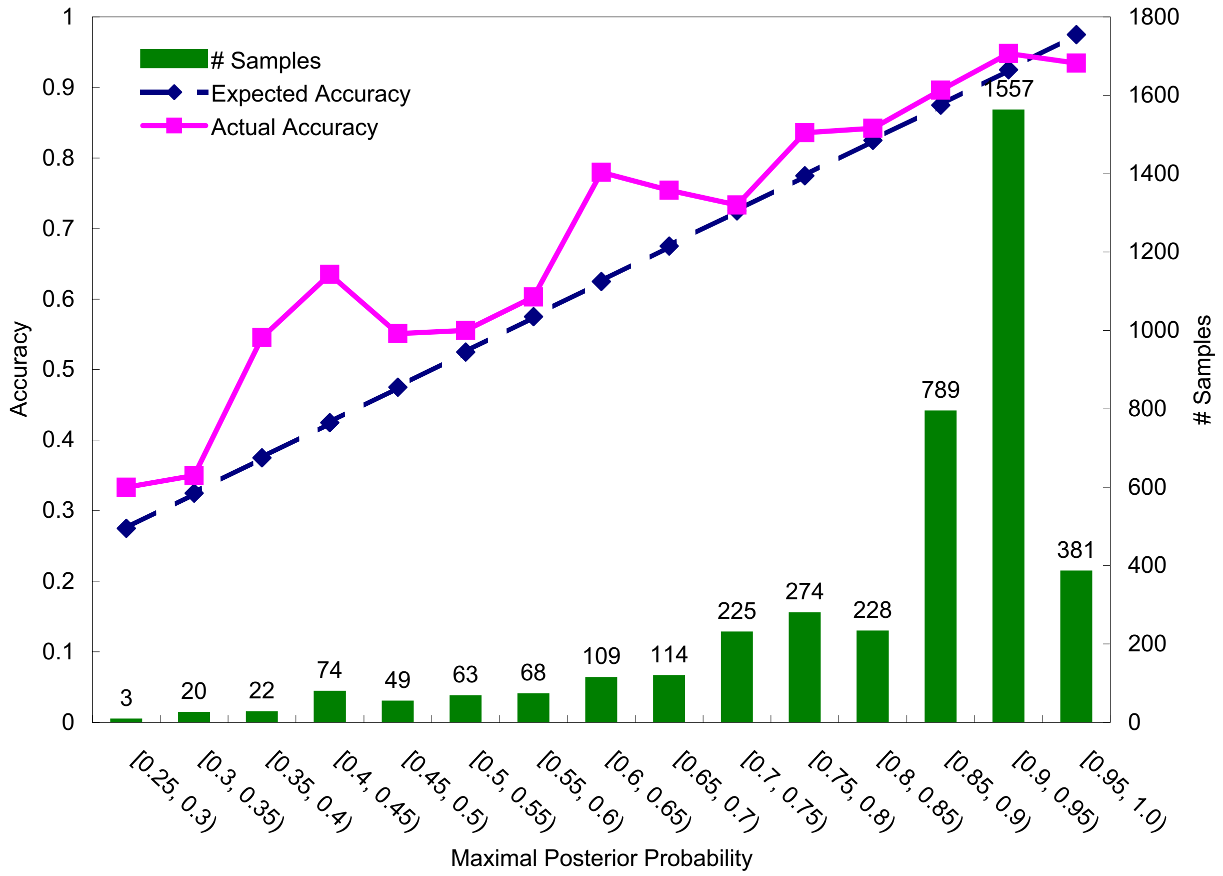

The actual accuracy rates are slightly higher than the estimated posterior probabilities.
